# High sugar content of European commercial baby foods and proposed updates to existing recommendations

**DOI:** 10.1111/mcn.13020

**Published:** 2020-08-30

**Authors:** Jayne Hutchinson, Holly Rippin, Diane Threapleton, Jo Jewell, Haidi Kanamäe, Kristin Salupuu, Margherita Caroli, Angelo Antignani, Lucienne Pace, Charlene Vassallo, Britt Lande, Christina Hildonen, Ana Isabel Rito, Mariana Santos, Mojca Gabrijelcic Blenkus, Eszter Sarkadi‐Nagy, Gergő Erdei, Janet E. Cade, Joao Breda

**Affiliations:** ^1^ Nutritional Epidemiology Group, School of Food Science and Nutrition University of Leeds Leeds UK; ^2^ Division of Noncommunicable Diseases and Promoting Health through the Life‐Course World Health Organization Regional Office for Europe Copenhagen Denmark; ^3^ Nutrition and Exercise Unit, Centre for Health Risks Prevention National Institute for Health Development Tallinn Estonia; ^4^ Department of Nutrition Research National Institute for Health Development Tallinn Estonia; ^5^ The European Childhood Obesity Group (ECOG) Rome Italy; ^6^ Department of Clinical Medicine and Surgery University of Naples Naples Italy; ^7^ Health Promotion and Disease Prevention Directorate Msida Malta; ^8^ Division Prevention and Public Health Norwegian Directorate of Health Oslo Norway; ^9^ Department of Food and Nutrition National Health Institute Dr.Ricardo Jorge (INSA) Lisbon Portugal; ^10^ NOVA National School of Public Health Universidade NOVA de Lisboa Lisbon Portugal; ^11^ National Institute of Public Health Slovenia Ljubljana Solvenia; ^12^ National Institute of Pharmacy and Nutrition Budapest Hungary

**Keywords:** commercial foods, complementary feeding, dietary guidelines, food packaging, infant food, nutrition policy, sugars

## Abstract

The aim was to determine whether commercial baby foods marketed within Europe (up to 36 months of age) have inappropriate formulation and high sugar content and to provide suggestions to update European regulations and recommendations as part of a nutrient profile model developed for this age group. The latter was produced following recommended World Health Organization (WHO) steps, including undertaking a rapid literature review. Packaging information from countries across the WHO European region was used to determine mean energy from total sugar by food category. The percentage of products containing added sugar and the percentage of savoury meal‐type products containing pureed fruit were also calculated. A total of 2,634 baby foods from 10 countries were summarised: 768 sold in the United Kingdom, over 200 each from Denmark (319), Spain (241), Italy (430) and Malta (243) and between 99–200 from Hungary, Norway, Portugal, Estonia and Slovenia. On average, approximately a third of energy in baby foods in these European countries came from total sugar, and for most food categories, energy from sugar was higher than 10%. Use of added sugars was widespread across product categories, with concentrated fruit juice most commonly used. Savoury meal‐type purees did not contain added sugars except in United Kingdom and Malta; however, fruit as an ingredient was found in 7% of savoury meals, most frequently seen in UK products. Clear proposals for reducing the high sugar content seen in commercial baby foods were produced. These suggestions, relating to both content and labelling, should be used to update regulations and promote product reformulation.

**Key messages**

- Product data collated from 10 countries across the WHO European region show that most categories of commercial baby foods (up to 36 months) have high sugar contents, including apparently savoury‐type foods. Use of added sugars and sweetening agents, particularly concentrated fruit juice and pureed fruit, is widespread in commercial baby foods marketed in Europe.
- Infants and young children are being exposed to high levels of added, free and total sugar from commercial baby foods, despite WHO's recommendation to limit free sugars in foods for this age group and to reduce total sugar to <10% of energy for older children. Such sweet foods may contribute to sweet taste preference development leading to excess energy intake and tooth decay in later years.
- To support existing health policy for young children, political and organisational commitments are needed to facilitate updated regulations and widespread baby food reformulation across Europe.
- We address the regulatory gap and go beyond previous research by providing clear proposals for reducing sugar in commercial baby foods, as part of a WHO‐led process of developing a nutrient profile model tool to end the inappropriate promotion of foods for infants and young children.
- The proposals include prohibiting added sugars, limits on use of pureed fruit in some food categories, limiting total sugar content of 'savoury' snacks and banning sweet snacks. Proposed labelling requirements and restrictions include improving labelling of total sugar and total fruit content, banning misleading product names and claims, and restricting the upper age limit of heavily pureed foods.

## 1 INTRODUCTION

The early life period from conception until 2 years of age—the so‐called first 1,000 days of life—is a critical window during which the environment, including nutrition, can have a profound influence on the development of the foetus, infant and child (Woo Baidal et al., 2016) and also the risk of disease in later life (Hanson & Gluckman, 2014; Langley‐Evans, 2015). For instance, breastfeeding for less than 6 months, which is common in Europe, is linked to higher risk of childhood obesity (Rito et al., 2019). Other potential risk factors for childhood obesity in relation to complementary feeding during infancy include rapid weight gain, premature introduction of solids and potential interactions with the gut microbiome (Huh, Rifas‐Shiman, Taveras, Oken, & Gillman, 2011; Mameli, Mazzantini, & Zuccotti, 2016; Pihl et al., 2016; Woo Baidal et al., 2016). Additionally, observational longitudinal analyses indicate that dietary habits and taste preferences formed at young ages may persist into later years (Skinner, Carruth, Bounds, & Ziegler, 2002). The World Health Organization (WHO) recommends that infants should be breastfed exclusively for the first 6 months of life to achieve optimal growth, development and health (WHO, 2001). Thereafter, they should receive nutritionally adequate and safe complementary foods while breastfeeding continues up to 2 years or beyond (WHO, 2002). However, evidence indicates that food and drinks high in fats, sugars and salt are being marketed for consumption by infants and young children (Crawley & Westland, 2017; Maslin & Venter, 2017).

The 2015 WHO guideline on sugar intake strongly recommends reducing free sugar intake, throughout the life course, to below 10% of total energy and preferably below 5% of total energy intake (WHO, 2015). The guidelines are supported by robust evidence of a relationship between sugar intake and dental caries and links between free sugars or sugar sweetened beverage intake and weight gain (Breda, Jewell, & Keller, 2019). In addition, excessive sugar intake is associated with increased risk of non‐communicable diseases (Breda et al., 2019). Although these WHO guidelines do not focus specifically on infants and young children, some European countries recommend limiting added sugars for this age group (Grammatikaki, Wollgast, & Caldeira, 2019) and the American Heart Association states that added sugar should be avoided under 2 years (Vos et al., 2017). Across Europe, added sugars contribute 11%–17% of total energy intake in children (Azaïs‐Braesco, Sluik, Maillot, Kok, & Moreno, 2017). Against this backdrop, evidence is growing that some commercial baby foods contain very high amounts of sugar. About half (53%) of commercial baby foods examined in Canada contained over 20% energy from sugar; baby desserts, teething biscuits and fruit/yogurt snacks and cereals had the greatest energy from sugar (Elliott, 2011). Sweet products dominate the UK market for early complementary feeding (labelled as 4+ or 6+ months) (Crawley & Westland, 2017; Garcia, Raza, Parrett, & Wright, 2013), with many products, even savoury meals, deriving much of their energy content directly from free sugars, via added fruit juice, pureed fruit or sweet vegetables. The intense pureeing process used to produce smooth baby foods liberates intrinsic sugars from fruit and vegetable cell walls (Crawley & Westland, 2017; SACN, 2016), creating readily available free sugars. Furthermore, front‐of‐pack names of products, such as those sold in the United Kingdom, are often not representative of the main ingredients and may mislead consumers about the relative amounts of different foods in products (Crawley & Westland, 2017). Evidence of inappropriate product labelling in many locations across Europe is sparse.

In 2016, the World Health Assembly approved the WHO guidance on ending the inappropriate promotion of foods for infants and young children (resolution WHA69.9), which called for restrictions on marketing of commercial complementary foods so that they do not interfere with breastfeeding, contribute to obesity and non‐communicable diseases, create a dependency on commercial products or mislead caregivers (e.g., via health and nutrition claims), whilst ensuring that products do not contain high sugar, fats or salt (WHA, 2016; 2016). Existing European Commission (EC) and Codex guidelines are intended to ensure that baby foods are safe and adhere to minimum or maximum nutrient thresholds (FAO/WHO, 2017). However, the high added, free or total sugar content of commercial baby foods is not fully addressed in existing guidelines. To address such issues, the WHO guidance called for the development of nutrient profile models to guide decisions about which foods are inappropriate for promotion to infants and young children and ensure that permitted products are promoted appropriately, focusing particularly on avoiding free sugars and salt (EC, 2006; WHA, 2016). Nutrient profiling is the science of classifying foods according to their nutritional composition for reasons related to preventing disease and promoting health.

This research forms part of a larger WHO‐commissioned project to prepare a nutrient profile model that aims to categorise and identify whether or not commercially available foods in the WHO European region are suitable to be marketed for infants and young children aged 6–36 months. This model details nutrient thresholds and labelling requirements, by food category, to support the establishment and amendment of effective legal and policy measures in European countries to avoid inappropriate promotion of commercial baby foods (WHO Regional Office for Europe, 2019). Here, we report whether baby foods marketed in countries across the WHO European region are inappropriate with respect to their sugar content and whether packaging and product names are misleading in this respect. Based on these findings, we provide suggestions to update European regulations and recommendations in relation to sugar and sweet ingredients; these proposals were incorporated into the recently published draft nutrient profile model for infants and young children (WHO Regional Office for Europe, 2019).

## 2 METHOD

### 2.1 Developing the proposals to reduce sugar content of commercial baby foods as part of a nutrient profile model

Proposals to improve the quality of baby foods, including reducing sugar content, were incorporated into a draft nutrient profile model; this was developed by following recommended WHO steps, using the advice reported from the 2010 WHO nutrient profiling technical meeting (WHO, 2011). This was an iterative and collaborative process, which involved the following:

1. Making reference to the International Code of Marketing of Breast‐milk Substitutes, existing EC directives, Codex standards for baby foods, relevant WHO guidance and existing WHO Regional Office for Europe nutrient profile model for children over 36 months;
2. Undertaking a rapid literature review of the issues related to complementary feeding and marketing of baby food such as age of solid food introduction, development of taste preferences and sweet and savoury flavours in baby food, the role of food texture, purees and pouches, nutritional quality, health implications relating to overweight and obesity and tooth decay, and marketing and packaging claims;
3. Using descriptive analysis of back‐of‐packet information of products marketed for infants and young children up to 36 months in Denmark, Spain and the United Kingdom (reported here) to establish food categories and propose nutrient content thresholds and using data from a further seven countries (reported here) to assess the suitability of the categories and thresholds in a provisional nutrient profile model.
4. Based on the above information and data (collected in Steps 1–3), and using feedback from researchers and experts around Europe, amendments were made to the food categories, nutrient thresholds and labelling requirements to produce those detailed in the published draft nutrient profile model (WHO Regional Office for Europe, 2019). The proposals developed relating to sugar are reported here.

### 2.2 Data collection

Food label data for foods labelled as suitable for complementary feeding for infants and young children up to 36 months of age (referred to as baby foods in this document) were collected from 10 countries: UK, Italy, Denmark, Malta, Spain, Slovenia, Estonia, Portugal, Hungary and Norway. Product food categories were established using the UK data as the first and most comprehensive database reviewed and then amended using Danish and Spanish data where necessary. The product data from baby foods on sale in the United Kingdom in 2016/2017 were extracted from a commercial online repository of packet‐label product information. Products on sale in Denmark in 2016/2017 and in Spain in 2017 were collected primarily from manufacturer or supermarket websites. Nutrient thresholds and labelling requirements were suggested for these food categories as part of the development of a draft WHO nutrient profile model for infants and young children aged 6–36 months in the European region.

Next, WHO invited researchers from seven further European countries to compile a list of approximately 100–200 baby foods to be representative as far as possible of products on their domestic markets in 2018. Products in as many of the food categories as possible were included to pilot test the nutrient profile model. Instructions were provided, and involvement of nutrition experts from the national institutes of public health and academic institutions was recommended. Table S1 in the supplementary file provides a list of brands included. Notes to S2 explain the definition of products marketed as baby foods for infants and young children up to 36 months of age. The product sample from Norway (*N* = 99) was a convenient sample and is not representative.

The taxonomy established from UK, Danish and Spanish data (shown in Table S2) was used to categorise data from the seven remaining countries. These data were used to verify the suitability of the categories for the nutrient profile model across Europe and used to compare product composition across countries. Each country was provided with a spreadsheet template created by JH to record and summarise data. For each product, researchers were required to choose a product category from the spreadsheet drop‐down list and enter product name, ingredients, nutrient and packaging details from food label information. They also were required to indicate whether the product contained any of the specified added sugars (see below for list), showing this in separate columns for each type of added sugar. For each country, duplicate products were excluded, but products marketed in more than one country were included in multiple data sets.

Final checks and product coding amendments for each country were undertaken centrally by HR and JH from the University of Leeds. For each product, the product category chosen by the country researchers was checked alongside the listed ingredients to ensure that these met the product category description provided (as show in Table S2). The mean percentage energy from sugar for each product category calculated by the spreadsheet was checked and investigated further if unusual. The ingredient list for each product was double checked for added sugars. Amendments within spreadsheets were made where necessary, and HR liaised with the country researchers to inform and agree any changes.

### 2.3 Product categorisation and classification

Categories include dry instant cereals, various types of purees, meals with chunky pieces, snacks, confectionery and biscuits (product categories and definitions are provided in Table S2 along with details of product exclusions). Products containing vegetables and other carbohydrates, in addition to proteins meat, fish or cheese, were classified as savoury meals, even when they also contained some fruit or sweet root vegetables. Soft, wet spoonable products containing mostly dairy protein such as yogurt were classified as dairy.

The presence of added sugars (defined below) was taken from back‐of‐packet ingredient lists. For these analyses, added sugars were classed as fruit juice whether whole, concentrated or powdered (except lime, lemon or equivalent citrus juice used in small amounts as a preservative); sugar; sucrose; dextrose; fructose; maltose; any syrup; honey; barley malt/malted barley/malt extract; molasses; and artificial or natural zero/low‐calorie sweeteners. This definition extends the European Food Safety Authority (EFSA) definition of added sugars (European Food Safety Authority, 2010) (bottom of Table S4) by additionally including fruit juice (and derivatives) and honey, in line with the WHO definition of free sugar (WHO, 2015). Lactose was not classed as an added sugar because it is a component of milk, and some dry products are made using milk constituent parts rather than fresh milk and list these as separate ingredients. Pureed fruit was not classed as an added sugar for the purpose of this analysis [although the UK Scientific Advisory Committee on Nutrition (SACN) and Public Health England (PHE) consider blended, pulped, pureed or extruded fruit as free sugars, SACN, 2016; Swan, Powell, Knowles, Bush, & Levy, 2018, which is a logical extension and interpretation of the WHO definition; WHO, 2015]. Instead, the percentage of savoury meal products containing pureed fruit has been tabulated separately (bottom of table 2). Country data providers were also asked to note other sweet ingredients found in their products and to provide examples of misleading front‐of‐pack product names such as those that do not mention high proportions of free sugar ingredients.

### 2.4 Content analyses

The percentage energy from total sugar for each product was calculated from the total grammes of sugar and total energy per 100‐g product taken from the back‐of‐pack nutrient content list. For each product category, the mean total grammes of sugar per 100‐g product and the mean percentage energy from total sugar were then calculated and tabulated for each country. The percentage of products in each product category containing added sugar was also tabulated; these results, and the percentage energy from total sugar for each country, were displayed in charts when data were available for at least four products per category. The amount of added sugar was not provided on product labels and hence could not be collated. JH prepared the tables and figures collating the results of the 10 countries.

## 3 RESULTS

### 3.1 Total sugar

The manufacturer‐reported sugar contents of 2,634 baby food products from 10 countries were summarised; 768 were collected for the United Kingdom, over 200 each from Denmark (319), Spain (241), Italy (430) and Malta (243) and between 99–200 from Hungary, Norway, Portugal, Estonia and Slovenia (Table S3). In most countries, fruit purees were the most frequently reported item, but ‘dry instant cereals’ (category 1a) were the most common products in Spain (74/241) and Italy (75/430). Italy also listed a large number of ‘meat‐only purees’ (25/430) and ‘teething biscuits’ (27). The United Kingdom listed a large proportion of meals containing chunky pieces, often sold in trays or pots (89/768), and also ‘savoury snacks’ (79). Some country data sets listed few or no products in certain categories; these may be less common or simply not selected as a part of the sample.

**FIGURE 1:**
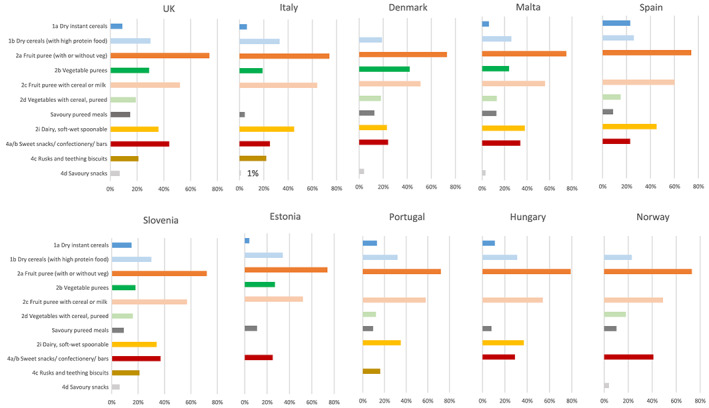
Mean percentage of energy from total sugar in baby foods marketed for <36 month olds by food category and country. No bar for a food category indicates fewer than 4 products in that category for that country were examined. Not all food categories are shown ‐ see Table 1

Analysis of the energy contribution from total sugars revealed that on average by country, products contained between 29% (Italy) to 44% (Hungary) of energy from total sugar (Table 1 and Figure 1), though for most food categories, the contributions were similar across countries, including for these two countries. Fruit purees (with or without vegetables) and fruit drinks had the highest percentage total sugar content, with the mean for each country being between 72%–79% and 68%–91%, respectively, whereas ‘vegetable‐only purees’ contained between 10%–42% sugar on average. The pureed and chunky savoury meals (i.e., categories 2e–h, 3a–b) were lower in sugar, but still derived a considerable proportion of calories from total sugar, with averages for United Kingdom, Denmark, Estonia and Malta all exceeding 10% energy from total sugar (see Table S4 for mean total grammes sugar per 100‐g product for each food category). Generally, dry products contained more sugar than purees. Products categorised as ‘sweet snacks, confectionery and bars’ contained on average between 23% (Spain) to 44% (United Kingdom) energy from total sugar. Average sugar contents of the ‘rusk and teething biscuits’ category were also high, being >20% in four of six countries that reported these. Approximately a third of energy in the ‘dairy’ and ‘dry cereal (with high‐proteinfood—powder milk or whey)’ categories was from total sugars (including milk sugars), and for dairy products, it was as high as 45% in Spain and Italy.

**TABLE 1:** Percentage energy from total sugar in commercial baby foods by category and country

Food category	United Kingdom	Italy	Denmark	Malta	Spain	Slovenia	Estonia	Portugal	Hungary	Norway
1a Dry instant cereals (%)	9	6	10^a^	6	23	15	4	13	11	‐
1b Dry cereals (with high protein food) (%)	30	33	19	26	26	30	34	32	31	23
2a Fruit puree (with or without vegetables) (%)	74	74	73	75	74	72	74	72	79	73
2b Vegetable purees (%)	29	19	42	24	10^a^	18	27	24^a^	‐	33^a^
2c Fruit puree with cereal or milk (%)	52	64	51	56	60	57	52	58	54	49
2d Vegetables with cereal, soft, wet spoonable (%)	19	‐	18	13	15	16	11^a^	12	2^a^	18
2e, 2f, 2g, 2h Savoury pureed meals (%)^b^	15	4	13	13	9	9	11	9	8	10
2i Dairy, soft, wet spoonable (%)	36	45	23	38	45	34	25^a^	35	37	38^a^
2j Meat‐only puree (%)	‐	0	‐	0	‐	0^a^	0^a^	‐	0^a^	‐
3a, 3b Tray/pot chunky meals (%)^b^	12	0	10^a^	11^a^	8^a^	8	16	8^a^	‐	‐
4a, 4b Sweet snacks and confectionery and bars (%)	44	25	24	34	23	37	25	42^a^	29	41
4c Rusks and teething biscuits (%)	21	22	‐	28^a^	‐	21	5^a^	16	‐	‐
4d Savoury snacks (%)	7	1	4	3	1^a^	6	4^a^	‐	‐	4
4e Fruit snacks (%)	61	‐	‐	93^a^	‐	62^a^	‐	‐	‐	‐
5a, 5bJuices and drinks (%)^b^	76	80	‐	‐	80^a^	85	68	89^a^	89	91^a^
Overall percentage from total sugar (%)	33	29	39	34	36	34	41	36	44	36
Number of products overall	768	414^c^	319	243	241	152	134	125	123	99

a Fewer than four products examined in the food category. See Table S3 for number of products in each category (‐ indicates no products examined).

b See Table S2 for description of food categories.

c Number of products examined in additional categories suggested by Italy: dry instant meat/fish *n* = 12; dry instant Vegetable *n* = 4.

### 3.2 Added sugar

**FIGURE 2:**
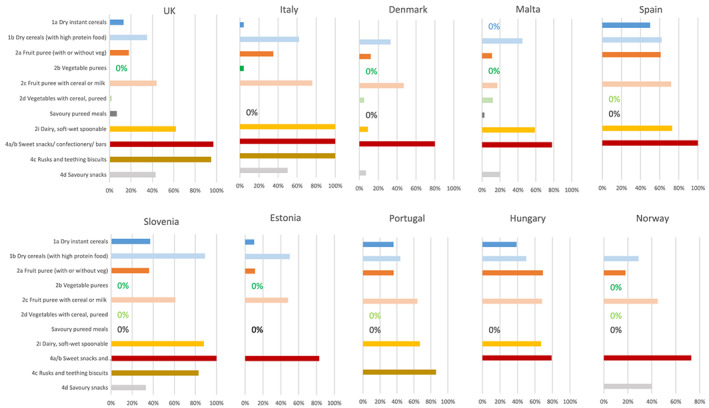
Percentage of products containing added sugars in baby foods marketed for <36 month olds by food category and country. 0% indicates no added sugar in products examined in that category. No bar, nor a %, indicates fewer than 4 products in that category for that country were examined. Not all food categories are shown ‐ see Table 2

On average by country, between 21% (Denmark) to 58% (Hungary) of products listed an ‘added sugar’ as an ingredient (Table 2 and Figure 2). Most baby foods in the ‘sweet snacks, confectionery and bars’ and ‘rusk and teething biscuits’ categories contained an added sugar. A high percentage of ‘fruit purees (with or without vegetables)’ contained added sugars in some countries, for example, Hungary (69%), Spain (61%), Portugal (36%), Slovenia (36%) and Italy (35%), and in most countries, added sugars were present in a high percentage of ‘fruit purees (with cereal or milk)’ and ‘dry cereals (with high‐protein foods)’. In some countries, a high percentage (30%–50%) of ‘savoury snacks’ contained added sugars, though numbers sampled were generally low. Conversely, most savoury meal products did not contain an added sugar, although some were found in UK products.

**TABLE 2:** Percentage of commercial baby foods containing added sugar by food category and country

Food category	United Kingdom	Italy	Denmark	Malta	Spain	Slovenia	Estonia	Portugal	Hungary	Norway
1a Dry instant cereals (%)	13	4	0^a^	0	50	37	10	36	39	‐
1b Dry cereals (with high‐protein food) (%)	35	62	33	45	62	89	50	44	50	29
2a Fruit puree (with or without vegetables) (%)	18	35	12	11	61	36	11	36	69	18
2b Vegetable purees (%)	0	4	0	0	33^a^	0	0	0^a^	‐	0^a^
2c Fruit puree with cereal or milk (%)	44	76	47	17	72	61	48	64	68	45
2d Vegetables with cereal, soft, wet spoonable (%)	2	‐	5	12	0	0	0^a^	0	0^a^	0
2e, 2f, 2g, 2h Savoury pureed meals (%)^b^	7	0	0	3	0	0	0	0	0	0
2i Dairy, soft, wet spoonable (%)	62	100	9	59	73	88	0^a^	67	67	50^a^
2j Meat‐only puree (%)	‐	0	‐	0	‐	0^a^	0^a^	‐	0^a^	‐
3a, 3b Tray/pot chunky meals (%)^b^	18	0	0^a^	50^a^	50^a^	0	0	50^a^	‐	‐
4a, 4b Sweet snacks and confectionery and bars (%)	97	100	80	78	100	100	83	100^a^	79	73
4c Rusks and teething biscuits (%)	95	100	‐	100^a^	‐	83	33^a^	86	‐	‐
4d Savoury snacks (%)	43	50	7	20	0^a^	33	0^a^	‐	‐	40
4e Fruit snacks (%)	22	‐	‐	0^a^	‐	0^a^	‐	‐	‐	‐
5a, 5b Juices and drinks (%)^b^	100	100	‐	‐	100^a^	86	100	100^a^	100	100^a^
Overall percentage listing an added sugar (%)^c^	28	38	21	24	44	49	23	42	58	36
Number of products overall	768	414^d^	319	243	241	152	134	125	123	99
Average proportion of savoury pureed meals that includes fruit (%)	15	0	7	7	0	0	11	0	6	9

a Fewer than four products examined in the food category. See Table S3 for number of products in each category (‐ indicates no products examined).

b See Table S2 for description of food categories.

c The following listed ingredients have been classed as added sugars and sweeteners for this analysis: sugar, (any) syrup, fruit juice concentrated/powder/or not (other than lemon or lime juice), molasses, malt extract, barley malt, malted barley extract, maltose, dextrose, fructose, glucose, sucrose, honey or low artificial or natural low‐calorie sweeteners.

d Number of products examined in additional categories suggested by Italy: dry instant meat/fish *n* = 12; dry instant vegetable *n* = 4.

The main type of added sugars varied somewhat across countries and products, but fruit juice was most common, even occurring in savoury snacks, particularly in the United Kingdom and Slovenia. The ingredient labelled ‘sugar’ or ‘sucrose’ was one of the main added sugars in all countries except for Denmark and Estonia. Malted extract or malted barley was found in around 10% of products in Italy, Spain and Slovenia, and likewise, honey and syrup were found in a similar proportion in the Spanish and Hungarian data sets, respectively.

### 3.3 Fruit purees in savoury meal products

On average across the countries, 7% of 469 savoury meal purees included fruit as an ingredient (bottom of Table 2). United Kingdom (15%) and Estonia (11%) had the highest percentage of savoury pureed meals listing a fruit. Although meals that contained a small proportion of fruit (<10%) often did not mention fruit on the front of pack, those containing a higher fruit proportion sometimes list this on front of pack, but this was not consistently done. No savoury meal products in the Spanish or Italian data sets contained fruit. For the other countries, fewer than 10% of savoury meals contained fruit and those that did tended to be from UK‐based manufacturers. In the United Kingdom, 21% of the 89 savoury meals with chunkier pieces contained fruit. The fruit content by weight was not labelled in all products.

### 3.4 Misleading product names and claims

Products using large proportions of fruit puree but not listing this in the front‐of‐pack product name, or listing it inappropriately, were found in many country data sets. For example, some pureed foods with apple as the largest ingredient had not stated this ingredient on the front of pack or had mentioned apple in the name after other ingredients. Other products were described as yogurt or cereal/milk based, but consisted mainly of fruit (examples are in Table S5, along with suggestions of more appropriate names). Additionally, products containing pureed fruit or even concentrated fruit juice often stated that they contained no added sugar. Although this is strictly true, these very sweet fruit‐based ingredients will significantly contribute to the total and free sugar content of these foods.

### 3.5 Resulting proposals to reduce sugar content of commercial baby foods

A number of changes to the content, labelling and marketing of baby food were proposed in relation to sugar as detailed in Table 3, after taking into account the rapid literature review, current regulations and recommendations, and the current content of baby food products in Europe. This table also provides some justification and references for the recommendations. The proposals include prohibiting added sugars, limiting the use of pureed fruit in some food categories, limiting total sugar content of ‘savoury’ snacks and banning sweet snacks. Additional labelling requirements and restrictions are also proposed such as improving labelling of total sugar and total fruit content, banning misleading product names and claims and restricting the upper age limit of heavily pureed foods to 12 months of age. Further details of the literature review and the steps taken to produce the proposals as part of the development of the nutrient profile model can be found in the WHO discussion document (WHO Regional Office for Europe, 2019).

**TABLE 3:** Proposals to improve quality of baby foods and reduce total sugar intake in infants and young children aged 6–36 months

Recommendation	Details and justification
1. Baby foods should not be marketed as suitable for children under 6 month of age	•In line with WHO recommendations that infants should be exclusively breastfed for the first 6 months of life to achieve optimal growth, development and health (WHO, 2001, 2002). Thereafter, they should receive nutritionally adequate and safe complementary foods while breastfeeding continues for up to 2 years of age or beyond (WHO, 2002). •To ensure breastfeeding practises are not undermined (WHO, 1981). •Promotion of products as suitable for infants under 6 months is a violation of the International Code on Marketing of Breastmilk Substitutes (WHO, 1981).
2. Prohibit *added sugars* and sweet agents in all baby foods	•Likely one of the simplest and most effective ways to reduce overall baby food sugar content. •Aligns with WHO, governmental and other organisational policy and recommendations to reduce energy intake from total sugar and reduce added or free sugar intake in young children (Crawley & Westland, 2017; Grammatikaki et al., 2019; Vos et al., 2017; WHA, 2016).
3. Extend all definitions of *added sugars* to include fruit juice	•Fruit juice is not currently included in the EFSA definition of added sugars (European Food Safety Authority, 2010). •Fruit juice and fruit juice concentrate are widely used in baby foods and have high free sugar contents (Grammatikaki et al., 2019). •First Steps Nutrition Trust in the United Kingdom recommends phasing out products sweetened with fruit juice (Crawley & Westland, 2017).
4. Limit use of pureed fruit, particularly in savoury foods, yogurts and other desserts (e.g., ≤5% of total weight)	•As recommended by the First Steps Nutrition Trust in the United Kingdom (Crawley & Westland, 2017), this would reduce total exposure of infants and young children to intrinsic sugars liberated from fruit and vegetable cell walls. •Pureed fruit and concentrated fruit purees are used in high proportions in many products. Although they may technically adhere to many dietary guidelines and may not contain ‘added sugars’, they are rich in intrinsic free sugars, which is equally likely to contribute to taste preference development, excess energy intake or tooth decay (Dunford, Louie, Byrne, Walker, & Flood, 2015). •Products for early introduction of solid foods are predominantly smooth and sweet blends/purees and rarely include single food flavours or bitter vegetables; therefore, many may not meet the infants' need for exposure to a variety of textures, single flavours, bitter flavours and other non‐sweet foods (Fewtrell et al., 2017; Public Health England, 2018).
5. Reduce the proportion of very smooth pureed products available	•Very smooth baby foods (often sold in pouches) are very popular but have limited textures, may have high water contents (i.e., low nutrient/energy density) and/or high free sugar contents. Frequent selection of low‐quality foods (i.e., low energy density or high sugar content) may not provide the appropriate supplementary nutrition that is required for healthy growth and development (WHO Regional Office for Europe, 2019). •They may negatively influence children's learning about food texture (Fewtrell et al., 2017; Public Health England, 2018). At the start of food introduction, premashed foods enable efficient nutrient uptake for infants who are not yet able to manipulate foods in their mouth, but they are not required as chewing skills develop (Cichero, 2016). •Increasing the proportion of more textured products for infants under 9 months old may have additional benefits related to later food acceptance (Coulthard, Harris, & Emmett, 2009).
6. Introduce front‐of‐pack upper age restrictions for heavily pureed and very smooth products intended as weaning foods (e.g., suitable for age 6–12 months)	•Pureeing changes the flavour and appearance of foods, making them less recognisable, which may lead to overeating, as foods can be rapidly swallowed by older infants and young children without chewing. •Decrease overreliance on these very sweet and smooth foods for young children who should have developed chewing ability.
7. Phase out pouches for pureed foods with spouts and add pack labelling to prevent infants and young children from sucking from spouts	•Spouts are an easy delivery system for freely available sugars, discourage development of chewing skills and may enable young children to consume large volumes of sugar per eating episode (Koletzko et al., 2018). •Some manufacturers directly encourage caregivers to allow infants and young children to suck from pouches.
8. Improve product labelling for total sugar and total fruit contents (e.g., front‐of‐pack flags for high total or free sugar content and back‐of‐back ingredient information such as the total fruit content)	•Caregivers must be able to readily identify products with high sugar contents and compare similar products when making purchasing choices. •The high proportion of fruit ingredients is often not made clear on pack labels, and the proportion of fruit in some foods with high sugar content is not listed.
9. Ban misleading labelling and claims relating to sugar contents or product healthiness	•Baby food products should not include claims or statements that imply a low or ‘healthy’ sugar content such as ‘no added sugars’ or ‘contains only naturally occurring sugar’ (Crawley & Westland, 2017). •Health symbols, for instance ‘tooth friendly’ symbols found on children's foods in Europe, should not be used (Hieke et al., 2016). •Many products on the market have misleading names or include promotional statements that imply superiority of commercial products over home‐prepared foods.
10. Front‐of‐pack product names must be representative of primary ingredients	•The order of ingredients in products names often implies lower content of cheaper and sweeter ingredients (e.g., apple or banana) in place of more expensive fruits, vegetables (with lower sugar content) or protein and dairy foods. •Substantial fruit content in a product must be apparent in the front‐of‐pack product name (see Table S5 for further details).
11. Suppress the promotion of dessert‐type foods in the infant diet as a social norm	•A UK report recommends that dessert foods should not be introduced until 10 months (Crawley & Westland, 2017); instead, the focus should be on providing breakfast and two savoury meals, in order for nutrient‐rich foods to be provided as first foods around 6 months, when the volume of food intake will be low (Crawley & Westland).
12. Ban the marketing of fruit drinks and juices, confectionery and sweet snacks to infants and young children	•This is in line with the WHO nutrient profile model for children over 3 years of age (WHO Regional Office for Europe, 2015). •Aligns with WHO, governmental and other organisational policy and recommendations to reduce energy intake from total sugar and reduce added or free sugar intake in infants and young children (Crawley & Westland, 2017; Grammatikaki et al., 2019; Vos et al., 2017; WHA, 2016).
13. Limit total sugar content of dry savoury snack foods to ≤15% energy (currently marketed to older infants and young children under 36 months)	•This will prevent high sugar contents of foods that from description appear savoury in nature. •Aligns with WHO, governmental and other organisational policy and recommendations to reduce energy intake from total sugar and reduce added or free sugar intake in infants and young children (Crawley & Westland, 2017; Grammatikaki et al., 2019; Vos et al., 2017; WHA, 2016).

*Note.* Further details, justification and the process of nutrient profile model development in conjunction with the WHO regional office for European are provided in the WHO Discussion/Consultation document on commercial baby foods and the proposed WHO nutrient profile model for infants and young children up to 36 months of age: WHO Regional Office for Europe (2019) ‘Ending inappropriate marketing of commercially available complementary foods for infants and young children aged up to 36 months.’ Copenhagen, WHO Regional Office for Europe. http://www.euro.who.int/en/health‐topics/disease‐prevention/nutrition/publications/2019/ending‐inappropriate‐promotion‐of‐commercially‐available‐complementary‐foods‐for‐infants‐and‐young‐children‐between‐6‐and‐36‐months‐in‐europe‐2019.(WHO Regional Office for Europe, 2019).

Abbreviation: SACN, UK Scientific Advisory Committee on Nutrition

## 4 DISCUSSION

This research shows that around a third of total energy in commercial baby foods examined in each of the 10 European countries comes from sugar. This is high and goes against the existing WHO recommendation to limit free sugars in foods for this age group and far exceeds WHO recommendations for older children of <10% of their total energy intake (WHO, 2015). The mean total sugar contribution in most food categories was over 10%, even in savoury pureed meals in United Kingdom, Denmark, Malta and Estonia, which may mislead caregivers. Added sugars are widespread across many of the product categories, and a substantial proportion of savoury meals contains pureed fruit, particularly in the United Kingdom. Given the nature of the products (highly macerated, fruit puree based), the majority of these sugars can be considered free sugars. The total added and free sugar contents of baby foods are of great concern because sugar intake is linked to later health status, including development of dental caries, weight gain and increased risk of non‐communicable diseases, such as type 2 diabetes, cardiovascular diseases, some cancers and non‐alcoholic fatty liver disease (Breda et al., 2019; Greenwood et al., 2014; Keller, Heitmann, & Olsen, 2014). A focus on improving the sugar content of baby foods should therefore be high priority in governmental and organisational policy, as well as in manufacturing practice, to ensure that the very young are not exposed to foods that are unnecessarily sweet, on a regular basis. A series of guidelines to improve commercial baby food quality and reduce sugar contents is proposed here (details and justifications in Table 3).

Our findings of high total added or free sugars in commercial baby foods are concordant with other European studies. In a UK study of infant foods marketed in 2010–2011, 65% of products were identified as sweet, and the total sugar content of soft, wet spoonable ready‐made products equated to about a third of energy, with over 10% in savoury meals and about 20% in dry finger foods and snacks (Garcia et al., 2013). One German study reported added sugars in almost a quarter of products (Hilbig, Foterek, Kersting, & Alexy, 2015). A Portuguese study found that infant cereals have the highest total sugar content (29%–36%) of all ready‐to‐eat cereals (Rito et al., 2019). Using data from the Mintel Global New Products Database (Mintel GNPD), a recent EC report found that added or free sugars (using a slightly different definition that included lactose) are widely used in most baby food subcategories in European Union (EU) countries (Grammatikaki et al., 2019).

## 5 POLICY AND GUIDELINES

Of the 10 countries reported here, Italy, Malta, Portugal, Slovenia, Spain, Estonia and United Kingdom currently do not have sugar recommendations specifically for young children below 2 years of age in terms of percentage of energy from sugar. However, for all age groups, the recommend sugar intake is <5% of total energy in Estonia and < 10% in Hungary and Norway; Denmark recommends <10% energy up to 24 months of age. Many products marketed for infants and young children exceed these thresholds. A recent EC compilation reported that most EU countries recommend limiting added sugar intake in foods marketed for under 3 year olds, but few include a quantitative recommendation and half do not have recommendations regarding total and free sugars (Grammatikaki et al., 2019). The European Parliament's rejection of the EC proposal to retain the current infant food regulations (EC Directive 2006/125/EC) to allow cereal‐based products to contain up to 30% of energy from added sugars in revised EU regulation (EU No. 609/2013) demonstrates growing awareness and concern (EC, 2006; European Parliament, 2013). Added sugars are difficult to monitor because the content or proportions are not provided on packaging. Additionally, EC regulations for ready‐to‐use baby foods (Annex II of 2006/125/EC) refer only to total carbohydrate restriction and only in juices or desserts and puddings (EC, 2006).

A further complication for policies and guidelines is that fruit puree is currently not explicitly included in the WHO definition of free sugars (WHO, 2015), despite its high free sugar content and frequent use. There is a strong case to consider the final product free sugar content when setting new recommendations for infants and young children. The UK Scientific Advisory Committee on Nutrition definition stipulates that milk sugars and sugar within fruit and vegetable cell walls may not be defined as free sugars (Buttriss, 2015). However, intense maceration and heat treatment used in production of commercial baby food purees liberate intrinsic sugars from fruit and vegetable cell walls (SACN, 2016). A UK study noted that relatively sweet fruits and vegetables were most commonly used in baby foods (apple, banana, tomato, mango, carrot and sweet potato) (Crawley & Westland, 2017; Garcia, McLean, & Wright, 2016). Products containing added fruit juice or pureed fruit and sweet vegetables currently are not required to state that ‘added sugars’ are present; thus, caregivers may think they are providing healthy foods, while inadvertently reinforcing preferences for sweet foods (Garcia et al., 2016). Such sweet foods may condition hard‐to‐break habits (Thow & Hawkes, 2014), and infants' innate predisposition for sweet tastes (Ventura & Worobey, 2013) can easily be exploited by food companies (Thow & Hawkes, 2014). Evidence that higher intake of commercial foods in infancy is associated with greater sugar intake in later childhood supports the hypothesis that commercial foods in early infancy facilitate later life choices for sweeter foods (Foterek et al., 2016). Furthermore, commercial products lack diversity in terms of texture and taste, factors which may be important in establishing eating habits and preferences among older children (Le Révérend, Edelson, & Loret, 2013; Ventura & Worobey, 2013). However, understanding the influence of complementary feeding on later dietary habits and health is complex and often limited by observational evidence.

A comprehensive approach to policy across Europe is needed to support reduced sugar intake for infants and young children (Thow & Hawkes, 2014), and commercial manufacturers must be required to support such aims to protect this vulnerable demographic. Governments require evidence‐based guidance in order to steer food manufacturers to improve the quality of their products and provide unambiguous information on packets that will neither mislead consumers nor undermine public health recommendations. The recommendations in the WHO proposed nutrient profile model for infants and young children (parts of which are shown here in Table 3 in relation to sugar) were developed to guide stakeholders; the nutrient profile model may be modified for national use to account for differences in food culture, marketing and regulatory environments (WHO Regional Office for Europe, 2019). In some countries, a mandatory, rather than a voluntary, approach will likely be needed, and current regulations relating to commercial baby foods will need to be modified in such cases. Further considerations in applying the sugar proposals can be found in supporting information S6.

## 6 STRENGTHS AND WEAKNESSES

Strengths lie in the extensive assessment of currently available products in geographically dispersed parts of Europe and provision of practical and evidence‐based proposals to reduce sugar content and improve quality of baby foods. Total sugars are reported in terms of percentage energy, and the data sets include countries (Malt and Slovenia) not reported in recent contemporary European research (Grammatikaki et al., 2019). The product categories were carefully constructed to align with existing EC regulations (2006/125/EC) (EC, 2006) and were pilot tested to be applicable across Europe, during the development of a WHO‐led nutrient profile model (WHO Regional Office for Europe, 2019). This work is therefore valuable for setting marketing and product guidance and informing policies in Europe in order to align with important health priorities for the very young.

The product summaries reported here were not sales weighted, meaning that the relative or total consumption of each product or category cannot be determined. In addition, sampling was not systematic, which may introduce a level of bias. However, consistent guidance was provided to each country that asked for their sample to be representative as far as possible of products on their domestic markets, while including foods in different categories. However, where the smallest samples were examined, for example, ≤ 150 from Norway, Hungary, Portugal and Estonia, these are unlikely to be representative of all products and some of the higher average sugar contents observed for these may result from this. Despite this limitation, clear patterns were evident by food category across all locations, such as the high proportion of energy derived from total sugar in many categories. Packet information was used, and results therefore rely on manufacturers' reported content, rather than on independent laboratory analyses; therefore, actual sugar content could be higher than reported. Furthermore, the amounts of free sugar, added sugar and sweet ingredients are not routinely provided on packs, limiting assessment. Although our proposals suggest that pureed fruits could be restricted, these have not been included in our definition of ‘added sugar’ as the use of pureed fruit is ubiquitous in baby foods. However, this potentially results in an underestimation of the proportions of commercial foods that are unnecessarily or inappropriately sweet. Product analysis is also not divided by age recommendations on packaging as these are applied at the manufacturers' discretion. The inclusion of honey and fruit juice in our broader classification of ‘added sugar’ somewhat limits direct comparison with other contemporary work that used the European Food Safety Authority definition (European Food Safety Authority, 2010) but more appropriately addresses the very sweet taste profiles and composition of products currently on the market.

In addition, there are limitations in the body of evidence used to support the development and implementation of policies to restrict the inappropriate marketing of baby foods, as there are relatively few high‐quality studies providing evidence of the direct impact and health consequences of consuming commercial baby foods. It is usually not possible to conduct double blind trials investigating infant feeding practices, or it may be considered unethical to allocate healthy infants randomly to a dietary regimen (SACN, 2018). Patterns of feeding evolve rapidly in infancy; therefore retrospective data collection is often involved, which will be susceptible to recall bias. Confounding by socio‐demographic factors may also be an issue in observational studies. Furthermore, additional high‐quality research comparing the nutritional quality and effects of home‐cooked foods to commercially available complementary baby foods is needed (Maslin & Venter, 2017). However, we feel that there is sufficient evidence on the importance of the first 1,000 days of life, the negative health effects of high sugar intakes on health and the content of baby foods in Europe to warrant limiting the promotion of baby foods with high sugar content to infants and young children. Indeed, frequent selection of products high in sugar, low in nutrient density, may not provide appropriate nutrition for growth and development (WHO Regional Office for Europe, 2019).

In conclusion, the sugar content of commercial baby foods across Europe is high and contrary to existing health guidelines to restrict sugar intake in this vulnerable group. Updated regulations are urgently needed to support and guide product reformulation. Sugars can be restricted in a variety of ways, but first, consultation with WHO Europe member states is required to support the establishment of effective legal and policy measures in order to avoid inappropriate formulation and packaging for baby foods.

## CONFLICTS OF INTEREST

Professor Janet E. Cade is a director of a University of Leeds spin out private company, Dietary Assessment Ltd., supporting the development of myfood24.

## CONTRIBUTIONS

JB, JJ, JH, DT, JC and HR provided initial ideas and structure for this paper. The product category taxonomy was carried out by JH and DT at the University of Leeds in collaboration with JJ at the WHO Regional Office for Europe. JJ provided data from Denmark. JH categorised the UK, Spain and Denmark data. HK, KS, MC, AA, LP, CV, BL, CH, MS, MGB, ESN and GE provided ingredient and nutrient data of baby foods from food product labels marketed in stores or from websites in their country. They also allocated the products to the categories. Final cleaning and categorisation amendments of data sets were done by JH and HR. The first manuscript drafts were prepared by JH, HR, DT and JC, and the other authors reviewed the manuscript. A literature review by DT informed the manuscript.

## Supporting information


**Table S1**. Brands included in analyses
**Table S2.** Food categories for all foods marketed for infants and young children 6–36 months of age used in sugar analyses
**Table S3.** Number of products examined in study by food category by country
**Table S4.** Total g sugar per 100 g product in commercial baby foods by food category and country
**Table S5.** Product‐naming issues and proposed improved names for examples of commercial infant food products collected in 2016–18
**Supporting information S6.** Further considerations in applying proposals in Table 3 of the main document
