# Supplementary material for: High sugar content of European commercial baby foods and proposed updates to existing recommendations
Source: Matern Child Nutr. 2020 Aug 30;17(1):e13020. doi: 10.1111/mcn.13020 (PMC7729710; doi:10.1111/mcn.13020)
Supplement: Supplementary file 1 — Table S1. Brands included in analyses Table S2. Food categories for all foods marketed for infants and young children 6–36 months of age used in sugar analyses Table S3. Number of products examined in study by food category by country Table S4. Total g sugar per 100 g product in commercial baby foods by food category and country Table S5. Product‐naming issues and proposed improved names for examples of commercial infant food products collected in 2016–18 Supporting information S6. Further considerations in applying proposals in Table 3 of the main document [file MCN-17-e13020-s001.docx]

Supplementary materials S1:

| Brands included in analyses | | | | | | | | | |
| --- | --- | --- | --- | --- | --- | --- | --- | --- | --- |
| **UK** | **Italy** | **Denmark** | **Malta** | **Spain** | **Slovenia** | **Estonia** | **Portugal** | **Hungary** | **Norway** |
| Asda Little Angles, Annabel Karmel, Aptamil,  Babease,  Castlemil,  Clearspring, Cow & Gate, Ella’s Kitchen,  For Aisha,  Glenisk,  Goodness Gracious, Farley’s,  Fruit Bowl, Heavenly Tasty,  Heinz,  HiPP,  Little Dish, Kelkin,  Kiddylicious, Miniscoff,  Nestlé,  Nurture, Organix,  Pip and Pear, Piccolo,  Quinola Mothergrain, Yeo Valley | Coop,  Hipp  Humana,  Mellin,  Nestle,  Plasmon  Data collection in the UK, Denmark and Spain primarily informed development of the draft WHO Nutrient Profile Model (NPM) for infants and young children (IYC) 6-12 months in the European region. Product data from baby foods on sale in the UK in 2016/2017 were extracted from a commercial online repository of packet-label product information. Products on sale in Denmark in 2016/2017 and in Spain in 2017 were collected primarily from manufacturer or supermarket websites.  Collection in 2018 from seven other countries - Italy, Malta, Slovenia, Estonia, Portugal, Hungary and Norway - enabled pilot testing and refinement of the NPM for IYC. Instructions were provided to each site explaining the product information required and how this should be recorded. A database template was provided to summarise the data entered. They were asked to ensure that this list of 100-200 products was representative as far as possible of the range available on the market and to include products in as many food categories as possible. Involvement of nutrition experts from the national institutes of public health and academic institutions was recommended. The data informed our overall summaries of product sales across Europe but was not necessarily nationally representative for all countries. Data were collected from manufacturer or supermarket websites, or from packet-label photographs taken at retail outlets (in Malta). | Anglamark/Coop, Aria Baby & Me, Aurion,  Ella’s Kitchen,  Geia Food,  HiPP,  Holle,  Nestlé,  Organix,  Quinolababy, Semper | Curoe di Natura,  Ella’s Kitchen, Good Gout,  Heinz,  Hipp, Kiddylicious,  Maltova,  Milupa,  Nestle,  Organix,  Organu,  Piccolo,  Plasmon,  Tesco | Hero Baby, Blevit Ordesa,  HiPP,  Nestlé, Nutriben, Nutricia Almiron | Alete,  Alnatura,  Beaty baby  Bebivita  dm Bio  Frutek  Hipp  Julico,  Lino,  Mulupa,  Natur Aktiv Baby,  Nestle,  PICO,  Plasom | Ella’s Kitchen, Hipp,  Holle,  Põnn | Auchan,  Babybio,  Biobimbo,  Bledina,  Danone,  Hero,  Holle,  Nestle,  Nutriben,  ORganix,  Pingo Doce,  Smileat | Alete,  Detki,  DINKY,  dm Bio  Hame,  Hipp,  Kecskemeti,  Kinella,  Milumil,  Nestle,  Numil,  Nutricia,  OVKO,  Univer | Bear Nibbles,  Eldorado,  Ella’s kitchen  Hipp,  Kiddyliciouos,  Mini Me,  Nestle,  Rema 1000,  Semper |

Supplementary materials S2:

Food categories for all foods marketed for infants and young children 6–36 months of age used in sugar analyses^ab^

|  | **Food category** | **Definition and examples** |
| --- | --- | --- |
| **1** | **Dry, powdered and instant cereal/starchy food** | |
| 1a | Dry or instant cereals/starch with or without naturally sweet foods | Dry rice, cereal, pulverized rusks or starchy root (at least 25% cereal and/or starch root content) with or without naturally sweet foods (e.g., dry fruit)  To be prepared for consumption with milk or other appropriate nutritious liquid (e.g., formula)  Includes dry instant-type porridges and dry breakfast cereals (e.g., puffed rice or cereal hoops), if marketed as suitable for infants and young children  Excludes wet ready-to-eat cereals |
| 1b | Dry or instant cereals/starch **with** an added high-protein food | Dry rice, cereal, pulverized rusks or starchy root (at least 25% cereal and/or starchy root content) with an added high-protein food (e.g., milk or whey powder) to be prepared for consumption with water or other appropriate protein-free liquid |
| **2** | **Soft–wet spoonable, ready-to-eat foods** | |
| 2a | Fruit purée with or without vegetables | ≥ 95% single or mixed fruit (or mixed with vegetables)  Includes fruit-only smoothie purée/drinks and any spoonable fruit-only or fruit-and-vegetable purée |
| 2b | Vegetable purée | ≥ 95% single or mixed vegetables or legumes  Excludes products containing any fruit |
| 2c | Fruit with cereal or milk products | Largest ingredient is single or total fruit, plus cereals or dairy  Includes foods such as smoothies with > 5% dairy or cereal, high-fruit breakfast foods (e.g., fruit-based breakfast rice/ porridge) and desserts (e.g., apple crumble or fruit-based baby rice)  Excludes fruit/vegetable-based purées with < 5% cereal or dairy, which are categorized as 2a or 2b |
| 2d | Vegetables with cereals or milk products | Puréed or semi-puréed vegetables/legumes with > 5% cooked weight in cereal (e.g., pasta, rice, barley), or a pseudocereal (e.g., quinoa, chia, buckwheat)  Includes savoury-type meals with cereals (e.g., pasta with tomato and courgette) or pseudocereal (e.g., butternut squash, carrot and quinoa) or with milk products (e.g., cauliflower cheese/ macaroni cheese)  Includes vegetable-based foods containing cheese, where cheese is not mentioned in the product name |
| 2e | Meal with cheese mentioned in the name | A puréed or semi-puréed meal containing vegetables, other carbohydrates and cheese (e.g., cheesy pasta with tomato and vegetables) |
| 2f | Meal with fish mentioned first (as food) in name of product | A puréed or semi-puréed meal containing vegetables, other carbohydrates and fish  Fish is mentioned as first food in product name (e.g., “Tasty fish pie” or “Salmon and pea risotto”) |
| 2g | Meal with meat or poultry or other traditional source of protein mentioned first (as food) in name of product | A puréed or semi-puréed meal containing vegetables, other carbohydrates and meat, poultry or other traditional source of protein, where the source of protein is mentioned as first food in product name (e.g., “Hearty beef hotpot” or “Chicken and potato pie”) |
| 2h | Meals with meat, poultry, fish, offal or other traditional source of protein (but not named as the first food in product name) | A puréed or semi-puréed meal containing vegetables, other carbohydrates and traditional source of protein, where the meat/protein is not listed as first food in product name (e.g., “Hearty shepherd’s pie”, “Cottage pie” or “Carrot, potato and lamb hotpot”) |
| 2i | Dairy with or without fruit or other naturally sweet foods | Foods with dairy as the largest main ingredient by weight (i.e., greater than the sum of total fruit or total grain ingredients) such as yogurt, fromage frais, custard, porridge or rice pudding, made with or without other naturally sweet foods such as fresh fruit, fruit juice or dried fruit (excluding honey and other added sugars) |
| 2j | Only meat or poultry in name of product | Puréed or semi-puréed poultry, where poultry is the only food listed in product name and constitutes the single largest ingredient (except water) |
| 2k | Only fish or other traditional source of protein in name of product | Puréed or semi-puréed fish or other traditional source of protein, where this is the only food listed in product name and constitutes the single largest ingredient (except water) |
| **3** | **Meals with chunky pieces** | |
| 3a | Meat, fish or other traditional source of protein-based tray or pot meal | Non-puréed soft meals containing chunky pieces of vegetables, legumes or other carbohydrates and meat, fish or other traditional source of protein (often sold in trays) |
| 3b | Vegetable-based tray or pot meal | Non-puréed soft meals containing chunky pieces of vegetables, legumes or other carbohydrates (often sold in trays) |
| **4** | **Dry finger foods and snacks** | |
| 4a | Sweet confectionery, sweet spreads and fruit chews | Confectionery includes: chocolate and other products containing cocoa; white chocolate; jelly sweets and boiled sweets; chewing gum and bubble gum; caramels; liquorice sweets; marzipan; sweetened or “yogurt”-coated fruit etc.  Sweet spreads: spreadable chocolate and any other sweet sandwich/toast topping such as jam, marmalade or honey and sweet nut spreads etc.  Fruit chews include any dried and processed fruit products such as fruit gums, bars or fruit strips/leathers/roll-ups (i.e., a dense chewy food made from pulped and dehydrated/dried fruit) |
| 4b | Sweet snacks and finger foods | Any sweet baked, fried, dried or dehydrated food intended to be eaten between meals with ≥ 15% energy from total sugar (≥ 2.5 g/100 kcal) is classed as a sweet snack or finger food  Any starchy food, fruit-based or vegetable-based product where the sugar content is < 15% of total energy may be classed as a savoury snack (category 4d)  Including foods such as: sweet pastries; croissants; cookies/biscuits; sponge cakes; wafers; fruit pies; sweet buns; chocolate-covered biscuits; cake mixes and batters; cereal or energy bars (i.e., cereal/ granola or muesli bars); and crisps/puff products made from fruit, vegetables or starchy foods (which may be coated in fat/oil) |
| 4c | Rusks and teething biscuits | Light, crumbly or twice-baked dry sweet biscuit or bread to be chewed for teething or softened with liquid |
| 4d | Savoury snacks and finger foods | Foods consisting of ≥ 95% single or mixed grains, rice, potato, nuts, seeds, fruits or vegetables, including popcorn and maize corn with total sugar content < 15% energy from total sugar (< 2.5 g/100 kcal)  Any product with ≥ 15% energy from total sugar is classed as a sweet snack (category 4b)  Includes foods such as savoury biscuits and pretzels, baked chips/crisps (e.g., potato, grain or other starchy food etc.), rice cakes coated in powdered fruit or vegetables, cereal bars and rusks made without added sugars |
| 4e | Fruit (fresh or dry whole fruit or pieces) | Includes fresh whole or peeled fruit (e.g., apple) and dried fruit (e.g., dry slices of plain apple, freeze-dried strawberries, raisins, dry apricots, prunes)  Excludes fruit pieces coated in sugar or oils/fats (e.g., banana chips, sweetened cranberries or yogurt raisins) |
| **5** | **Juices and other non-formula drinks** | |
| 5a | Single or mixed fruit juices | Drinks made using anything other than ≥ 95% whole fruit (or fruit and vegetables) including fruit/vegetable cell walls (i.e., not ≥ 95% blended fresh fruit (or fruit and vegetable) pulp (which are classified in category 2a))  Includes drinks made using concentrated or strained/sieved fruit (e.g., apple juice, orange juice)  Excludes smoothies/purées which are ≥ 95% whole fruit (or fruit and vegetables) (see category 2a) |
| 5b | Single or mixed vegetable juices | Drinks made using “modified” vegetable pulp (i.e., not ≥ 95% blended fresh vegetables)  Includes drinks made using concentrated or strained/sieved vegetables  Excludes vegetable purées made using ≥ 95% vegetables (see category 2b) |
| 5c | Other non-milk-based drinks | Includes ready made from cordials, energy drinks, ices, cola, lemonade, orangeade, other soft drinks, and mineral and/or flavoured waters (including aerated) with added sugars or sweetener |

These are the food categories included in the provisional nutrient profile model which was tested using data from seven European countries. After feedback these categories were amended to those reported in the published Nutrient Profile Model (WHO Regional Office for Europe, 2019)

^a^ Exclusions to the food categories:

- products not specifically marketed for children younger than 3 years of age
- vitamin and mineral food supplements, whether to be consumed as tablets/drops or added to foods at home (e.g., home fortification products such as micronutrient powders, lipid nutrient powders)
- products that function as breast-milk substitutes (i.e., formula milk, follow-on formula milk)
- products whose labels state that they are intended only for pregnant women, mothers or children older than 3 years

^b^Products considered to be marketed as foods complementary to breast milk or breast-milk substitutes as being suitable for this age group if they:

- are labelled with the words “baby”, “infant,” “toddler” or “young child”;
- are recommended for introduction at an age of less than 3 years;
- have a label with an image of a child who appears to be younger than 3 years of age or feeding with a bottle; or
- are in any other way presented as being suitable for children under the age of 3 years.

Supplementary materials S3

|  | Number of products examined in study by food category by country | | | | | | | | | |
| --- | --- | --- | --- | --- | --- | --- | --- | --- | --- | --- |
| Food Category | **UK** | **Italy** | **Denmark** | **Malta** | **Spain** | **Slovenia** | **Estonia** | **Portugal** | **Hungary** | **Norway** |
| 1a Dry instant cereals | 31 | 75 | 3 | 18 | 74 | 8 | 10 | 25 | 11 | - |
| 1b Dry cereals (with high protein food) | 48 | 13 | 45 | 20 | 8 | 19 | 4 | 16 | 12 | 17 |
| 2a Fruit puree (with or without vegetables) | 140 | 60 | 97 | 53 | 41 | 14 | 38 | 25 | 13 | 22 |
| 2b Vegetable purees | 33 | 23 | 10 | 17 | 3 | 6 | 11 | 1 | - | 3 |
| 2c Fruit puree with cereal or milk | 54 | 41 | 47 | 12 | 25 | 23 | 25 | 14 | 31 | 11 |
| 2d Vegetables with cereal, soft-wet spoonable | 43 | - | 21 | 8 | 5 | 8 | 2 | 4 | 1 | 5 |
| Savoury pureed meals (2e, 2f, 2g, 2h)† | 145 | 72 | 60 | 44 | 57 | 20 | 18 | 15 | 16 | 22 |
| 2j Meat only puree | - | 25 | - | 8 | - | 1 | 3 | - | 1 | - |
| Tray/pot chunky meals (3a, 3b)† | 89 | 4 | 1 | 2 | 2 | 7 | 6 | 2 | 0 | 0 |
| 4a/b Sweet snacks and Confectionery and bars | 39 | 6 | 15 | 32 | 7 | 6 | 6 | 3 | 14 | 11 |
| 4c Rusks and teething biscuits | 19 | 27 | - | 1 | - | 6 | 3 | 7 | - | - |
| 4d Savoury snacks | 79 | 4 | 15 | 10 | 2 | 9 | 1 | - | - | 5 |
| 4e Fruit snacks | 9 | - | - | 1 | - | 1 | - | - | - | - |
| Juices and drinks (5a, 5b)† | 13 | 10 | 0 | 0 | 2 | 7 | 6 | 1 | 15 | 1 |
| Above total products | 768 | 414* | 319 | 243 | 241 | 152 | 134 | 125 | 123 | 99 |
| † †See supplementary materials S2 for more information on food categories  *Number of products examined in additional categories suggested by Italy: Dry instant meat/fish n=12; Dry instant Vegetable n=4 | | | | | | | | | | |

Supplementary materials S4

|  | Total g sugar per 100 g product in commercial baby foods by food category and country | | | | | | | | | |
| --- | --- | --- | --- | --- | --- | --- | --- | --- | --- | --- |
| Food Category | **UK** | **Italy** | **Denmark** | **Malta** | **Spain** | **Slovenia** | **Estonia** | **Portugal** | **Hungary** | **Norway** |
| 1a Dry instant cereals | 8.9 | 5.1 | 9.1† | 5.2 | 21.9 | 14.6 | 4.0 | 12.9 | 11.7 | - |
| 1b Dry cereals (with high protein food) | 31.7 | 33.9 | 21.2 | 27.2 | 25.1 | 28.4 | 37.0 | 33.3 | 33.1 | 25.1 |
| 2a Fruit puree (with or without vegetables) | 10.8 | 11.6 | 10.8 | 11.6 | 12.3 | 10.6 | 10.9 | 11.5 | 11.3 | 11.6 |
| 2b Vegetable purees | 3.4 | 1.9 | 3.8 | 2.2 | 1.5† | 2.1 | 2.8 | 1.5† | - | 3.0† |
| 2c Fruit puree with cereal or milk | 9.5 | 11.9 | 9.8 | 10.8 | 11.7 | 9.5 | 9.9 | 11.1 | 8.8 | 9.4 |
| 2d Vegetables with cereal, soft-wet spoonable | 2.9 | - | 2.6 | 1.6 | 2.4 | 2.4† | 1.1† | 1.7 | 0.4† | 2.4 |
| Savoury pureed meals (2e, 2f, 2g, 2h)‡ | 2.4 | 0.8 | 2.1 | 2.0 | 1.5 | 1.6 | 1.8 | 1.5 | 1.1 | 1.8 |
| 2i Dairy, soft-wet spoonable | 7.8 | 10.9 | 4.2 | 8.4 | 11.1 | 7.2 | 4.6† | 8.1 | 7.4 | 7.9† |
| 2j Meat only puree | - | 0.0 | - | 0.1 | - | 0.0† | 0.0† | - | 0.1† | - |
| Tray/pot chunky meals (3a, 3b)‡ | 2.4 | 0.0 | 2.2† | 2.1† | 1.4† | 1.6 | 2.2 | 1.3† | - | - |
| 4a/b Sweet snacks and confectionery and bars | 38.6 | 26.0 | 23.7 | 25.7 | 24.3 | 34.7 | 24.4 | 34.7† | 27.6 | 34.5 |
| 4c Rusks and teething biscuits | 21.7 | 22.6 | - | 29.0† | - | 20.9 | 5.5† | 17.7 | - | - |
| 4d Savoury snacks | 7.5 | 0.7 | 3.9 | 2.8 | 0.4† | 6.0 | 4.5† | - | - | 3.9 |
| 4e Fruit snacks | 54.2 | - | - | 69.3† | - | 56.0† | - | - |  | - |
| Juices and drinks (5a, 5b)‡ | 5.8 | 11.0 | - |  | 9.7† | 5.2 | 7.3 | 10.2† | 7.8 | 6.4† |
| Mean total sugar content | 10.3 | 11.1 | 9.8 | 10.8 | 12.8 | 11.2 | 8.5 | 13.3 | 12.5 | 12.8 |
|  |  |  |  |  |  |  |  |  |  |  |
| *Number of products overall* | *768* | *414** | *319* | *243* | *241* | *152* | *134* | *125* | *123* | *99* |

†Fewer than 4 products examined in the food category. See supplementary materials for number of products in each category (- indicates no products examined).

‡ ‡See supplementary materials for more information on food categories

*Number of products examined in additional categories suggested by Italy: Dry instant meat/fish n=12; Dry instant Vegetable n=4

**Definitions of total sugars, free sugar and added sugar**

Total sugars are all monosaccharides and disaccharides. Manufacturers are required to list total sugar content on product packaging.

WHO defines free sugars as “monosaccharides and disaccharides added to foods and beverages by the manufacturer, cook or consumer, and sugars naturally present in honey, syrups, fruit juices and fruit juice concentrates” (WHO, 2015). The United Kingdom Scientific Advisory Committee on Nutrition (SACN) has expanded on the WHO definition of free sugars to stipulate exclusions for milk sugars and sugar within fruit and vegetable cell walls:(Buttriss, 2015)

Under this SACN definition lactose (milk sugar) when naturally present in milk and milk products and sugars contained within the cellular structure of foods (particularly fruits and vegetables) are excluded.

Public Health England’s practical interpretation of the definition of free sugars has clarified that blended, pulped, puréed or extruded fruit is considered free sugars, which is a logical extension and interpretation of the WHO definition (Swan, Powell, Knowles, Bush, & Levy, 2018).

The European Food Safety Authority (EFSA) defines “added sugars” as sucrose, fructose, glucose, starch hydrolysates (glucose syrup, high fructose syrup) and other isolated sugar preparations used as such or added during food preparation and manufacturing (European Food Safety Authority, 2010). Sugar alcohols (polyols) such as sorbitol, xylitol, mannitol, and lactitol, are usually not included in the term “sugars”.

Additionally sugars present in honey, syrups, fruit juices and fruit juice concentrates are often considered "added sugars".

Supplementary material S5. Product-naming issues and proposed improved names for examples of commercial infant food products collected in 2016-18*

| **Brand/producer (reported by country)** | **Target age** | **Front-of-pack product name** | **Ingredients** | **Main name issue(s)** | **Proposed front-of-pack name** |
| --- | --- | --- | --- | --- | --- |
| Cow & Gate (UK) | From 7 months | Apple and blueberry yogurt | Apple (59%), yogurt (contains **milk**) (20%), blueberry (9%), banana (8%), rice flour, Aronia juice from concentrate, vitamin C | Name implies yogurt is main ingredient but fruit content = 76%. | Apple with yogurt and blueberry  OR  Apple and blueberry with yogurt |
| Ella’s Kitchen (UK) | 4+ months | Red peppers, sweet potatoes + apples  [purée] | Organic apples 78%, organic red peppers 11%, organic sweet potatoes 11%, organic lemon juice concentrate (a dash) | Misleading order of foods in name. | Apple with red peppers and sweet potatoes  [purée] |
| Ella’s Kitchen (UK) | 6+ months | Berry yogurt  [yogurt] | Organic Greek-style yogurt (milk) 34%, organic apples 31%, organic bananas 22%, organic blackcurrants 7%, organic blueberries 5%, organic rice starch 1%, organic lemon juice concentrate (a dash) | Name and label implies yogurt is main ingredient but fruit content = 65% Main ingredient (apple) is not named on front of pack. | Apple with yogurt and berries  [fruit puree with yogurt]  OR  Mixed fruit with yogurt and berries  [fruit puree with yogurt] |
| Heinz (UK) | 4+ months | Strawberry, raspberry & banana purée | Fruit (100%, concentrated apple purée (79%), strawberries (8%), banana (8%), raspberries (5%), concentrated lemon juice), vitamin C | Main ingredient (apple) is not named on front of pack. | Concentrated apple purée with banana and raspberry |
| Piccolo (UK) | 6+ months | Cherry and yogurt with wholegrain oats | Apples (43%), whole milk yogurt (milk) (30%), cherries (24%), wholemeal oat flavour (gluten) (3%), vanilla powder | Main ingredient (apple) is not named on front of pack. | Fruity yogurt with wholegrain oats  OR  Apple and yogurt with cherries and wholegrain oats |
| Põnn (Estonia) | From 8 months | Organic chicken and pasta meal | Organic butternut squash, water, organic chicken meat (15%), organic apple purée, organic carrot, organic almonds (incl. wheat flour durum) (4%), organic spinach | Does not mention the largest ingredient butternut squash in the name, nor apple in the name but this is a larger ingredient than pasta. | Butternut squash, chicken and apple with pasta |
| Põnn (Estonia) | From 4 months | Apple-carrot puree | Apple purée (40%), banana purée (40%), carrot purée (20%) | Banana not mentioned in name but is twice the weight of carrot. | Apple, banana and carrot puree |
| Rema 1000 (Norway) | From 6 months | Økologisk grøt med havre og bygg, mango og kiwi / Organic porridge, oat, barley, mango, kiwi | Mango puré (25%), Banana puree (20%), Water (19.4%), Kiwipuré (19%), Pear puree (10%), Oat flour (3%), barley flour (3% ), lemon juice from concentrate | Fruit content is more than water and dry oats combined. | Fruit puree with porridge  OR  Mango, banana, kiwi and pear puree with porridge |
| Ella’s Kitchen (Malta and Denmark) | From 7 months | Punchy pork roast dinner with apple sauce | Organic apple sauce 22%, (water and organic apples), organic potato 20%, organic vegetable stock 17% (water and organic vegetables parsnips, carrots, leeks, onions, and swedes), organic carrots 12%, organic pork 10%, organic onions 8%, organic leeks 6%, organic green beans 4%, organic garlic <1%,organic mixed herbs <1% (sage, thyme, marjam, parsley)organic pepper corns <1% | The largest ingredient apple sauce is not named first. | Apple sauce, roast pork dinner |
| HIPP (Slovenia) | From 7 months | Fruit porridge, banana, peach in apple with BIO yogurt | Fruit 94% (apple 77%, banana 14%, peach 3%), shot yogurt 5%, shot yoghurt powder, vitamin C. | Does not contain oats so incorrect to name it porridge. | Apple, banana and peach with BIO yogurt |
| HIPP (Hungary) | From 5 months | Kecskemeti vegyes zoldseg csirkehussal /  Vegetables with chicken | Water, Carrot 20%, Peas 9%, Chicken meat 8%, Rice grist, Cauliflower 5%, Rapeseed oil 2%, rice-starch, ferric diphosphate | Carrot is not named; this sweet vegetable makes up more than 50% of the vegetable in the product. | Carrot and vegetables with chicken |
| HIPP (Denmark) | From 8 months | Kyllingerisotto med grøntsager & ærter/ Chicken rissotto with Vegetables and Peas | Carrot 20%, boiled rice 18%, water, skimmed milk, tomato, peas, chicken 8%, celery, onion, rapeseed oil 1.8%, herbs and spices (peppers). | Carrot the largest ingredient is not named, and should be the first named food rather than the chicken. | Carrot and vegetable risotto with chicken |
| Bledina (Portugal) | From 6 months | Peru Com Cenouras/ Turkey and Carrot | Vegetable 63% (carrots 60%, onions 3%), cooking water 14%, durum wheat semolina 14%, turkey 8%, vegetable oil (rape, sunflower), sea salt. | Carrot is the largest ingredient and should be the first named food rather than turkey. | Carrot with turkey |
| Coop (Italy) | From 6 months | Verdure miste/ Mixed vegetables | Vegetables 73% (organic carrots, organic potatoes, organic cauliflower, organic peas in varying proportions), cooking water, corn seed oil. Gluten free. | The relative proportions of the vegetables are not given but carrots may form the main proportion of the product. | Carrot, potato, cauliflower and peas |

*the names of some of these products may have changed since samples were collected

**Supplementary material 6: Further considerations in applying proposals in table 3 of main document**

The consequences of imposing product bans or ingredient and content limits across product types (table 3) should be considered carefully within the context of the overall marketing environment. To achieve sweet tasting foods, manufacturers may reformulate products to take advantage of loop-holes such as using lactose or formula milk components (e.g. inulin, oligosaccharide, maltodextrose - see last paragraph for content in baby foods examined), which are not currently defined as added sugar. Unpalatable snack foods rejected by infants and young children, who may be accustomed to sweet tastes, may result in caregivers providing foods of poorer nutritional quality, not marketed as suitable for infants and young children. Realistic guidelines must therefore be weighed against idealistic guidelines to ensure snacks foods marketed for infants and young children are palatable and also have superior nutritional quality to snacks intended for older children or adults. Nevertheless the WHO Regional Office for Europe nutrient profile model for older children states marketing of confectionery and sweet snacks to children should not be permitted (WHO Regional Office for Europe, 2015).

Similarly, labelling suggestions such as front-of-pack 'flags' or 'traffic lights' indicating high sugar content should be applied after consultation with caregivers and manufacturers to ensure the information can be clearly presented and is acceptable to target customers. Complications and misunderstanding of such flags may arise if different sugar content thresholds are applied to different product types; for example if a higher threshold is set for dairy foods which have a naturally higher sugar content from lactose. Alternatively, a more easily understood comparison across products may be to use an estimated percentage energy from free sugars for front-of-pack labels, which would align better with WHO guidelines. However, this will be dependent on availability of reliable estimates of free sugars which are not currently required on the nutrition label.

Commercially available complementary foods for infants aged between 6-12 months should aim to add more nutrients and energy to the diet than breast milk in terms of density (Crawley & Westland, 2017; Dewey, 2001). However, imposing restrictions on the use of fruit juice or limiting total sugar content may result in lower energy or lower nutrient-dense products, particularly for fruit-based purees. Savoury meals rich in starches and vegetables as well as good sources of fat and protein should be promoted in place of simple purees. The WHO discussion document found a substantial proportion of purees in the UK and Denmark currently have energy densities below 60 kcal/100 g (i.e. lower than the density of breast milk) due in part to some purees or their ingredients having a high water content (WHO Regional Office for Europe, 2019). Therefore sugar content restrictions may need to be accompanied by minimum energy density requirements. This could result in higher fat and protein content, but products would need to be appropriate for the intended age and in-line with existing regulations for fat and protein.

Although we have proposed a maximum age limit of 12 months for purees, this could be reduced to 10 months as the European Society for Paediatric Gastroenterology, Hepatology, and Nutrition (ESPGHAN) state that prolonged use of pureed foods should be discouraged and infants should be eating lumpy foods by 8 to 10 months at the latest (Fewtrell et al., 2017).

Other Sweet ingredients

Our research showed only a small number of dairy and pureed meals listed lactose in the ingredients. Sweet ingredients, often found in breast milk substitutes, such as oligosaccharides, maltodextrin and inulin, were also listed in a small number of products in some countries. However, in the UK maltodextrin was labelled as an ingredient in 77% of the 48 'Dry cereals with added high protein' (i.e. dried milk or whey protein), and inulin was labelled in 37% of 'Rusks/ teething biscuits'. In Spain maltodextrin or “dextrinomaltosa” was found in over half and oligosaccharides in many of the 74 other dry cereal products.

References

Buttriss, J. (2015). *Why 5%? An explanation of the Scientific Advisory Committee on Nutrition’s recommendations about sugars and health, in the context of current intakes of free sugars, other dietary recommendations and the changes in dietary habits needed to reduce consumption of free sugars to 5% of dietary energy*. Retrieved from

Crawley, H., & Westland, S. (2017). *Baby foods in the UK. A review of commercially produced jars and pouches of baby foods marketed in the UK*. Retrieved from <https://static1.squarespace.com/static/59f75004f09ca48694070f3b/t/5a93f885085229b264ff6086/1519646858256/Baby_Food_in_the_UK+_2017.pdf>

Dewey, K. G. (2001). Nutrition, Growth, and Complementary Feeding of The Brestfed Infant. *Pediatric Clinics of North America, 48*(1), 87-104. doi:<https://doi.org/10.1016/S0031-3955(05)70287-X>

European Food Safety Authority. (2010). Scientific Opinion on Dietary Reference Values for Carbohydrates and dietary fibre. *EFSA Journal, 8*(3), 1462.

Fewtrell, M., Bronsky, J., Campoy, C., Domellöf, M., Embleton, N., Fidler Mis, N., . . . Molgaard, C. (2017). Complementary Feeding: A Position Paper by the European Society for Paediatric Gastroenterology, Hepatology, and Nutrition (ESPGHAN) Committee on Nutrition. *64*(1), 119-132. doi:10.1097/mpg.0000000000001454

Swan, G. E., Powell, N. A., Knowles, B. L., Bush, M. T., & Levy, L. B. (2018). A definition of free sugars for the UK. *Public health nutrition, 21*(9), 1636-1638. doi:10.1017/S136898001800085X

WHO. (2015). *Guideline: Sugars intake for adults and children*. Retrieved from Geneva: <https://www.who.int/publications-detail/9789241549028>

WHO Regional Office for Europe. (2015). *WHO Regional Office for Europe nutrient profile model* Retrieved from (<http://www.euro.who.int/en/health-topics/disease-prevention/nutrition/publications/2015/who-regional-office-for-europe-nutrient-profile-model-2015>).

WHO Regional Office for Europe. (2019). *Ending inappropriate promotion of commercially available complementary foods for infants and young children between 6 and 36 months in Europe: A discussion paper outlining the first steps in developing a nutrient profile model to drive changes to product composition and labelling and promotion practices in the WHO European Region*. Retrieved from Copenhagen: <http://www.euro.who.int/en/health-topics/disease-prevention/nutrition/publications/2019/ending-inappropriate-promotion-of-commercially-available-complementary-foods-for-infants-and-young-children-between-6-and-36-months-in-europe-2019>
